# Supplementary figures and images for: Diverse methanogens, bacteria and tannase genes in the feces of the endangered volcano rabbit (Romerolagus diazi)
Source: PeerJ. 2021 Aug 17;9:e11942. doi: 10.7717/peerj.11942 (PMC8378336; doi:10.7717/peerj.11942)

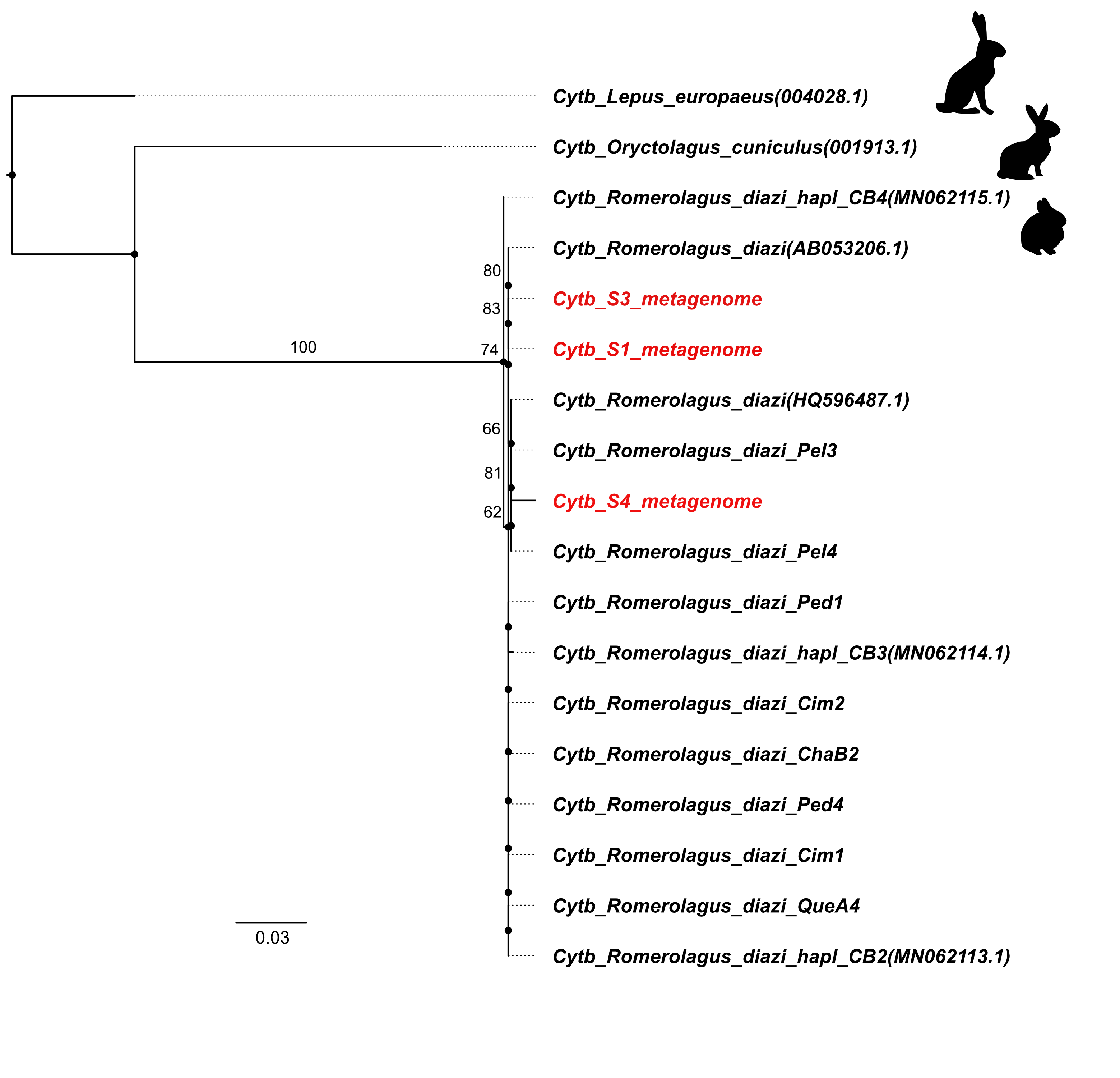

Supplement: Supplemental Information 1 — Cytb_S1_metagenome, Cytb_S3_metagenome and Cytb_S4_metagenome correspond to the mitochondrial sequences obtained from metagenomes. Cytochrome b mitochondrial sequences from Lepus europeus and Oryctolagus cuniculus were used as outgroups. [file peerj-09-11942-s001.png]

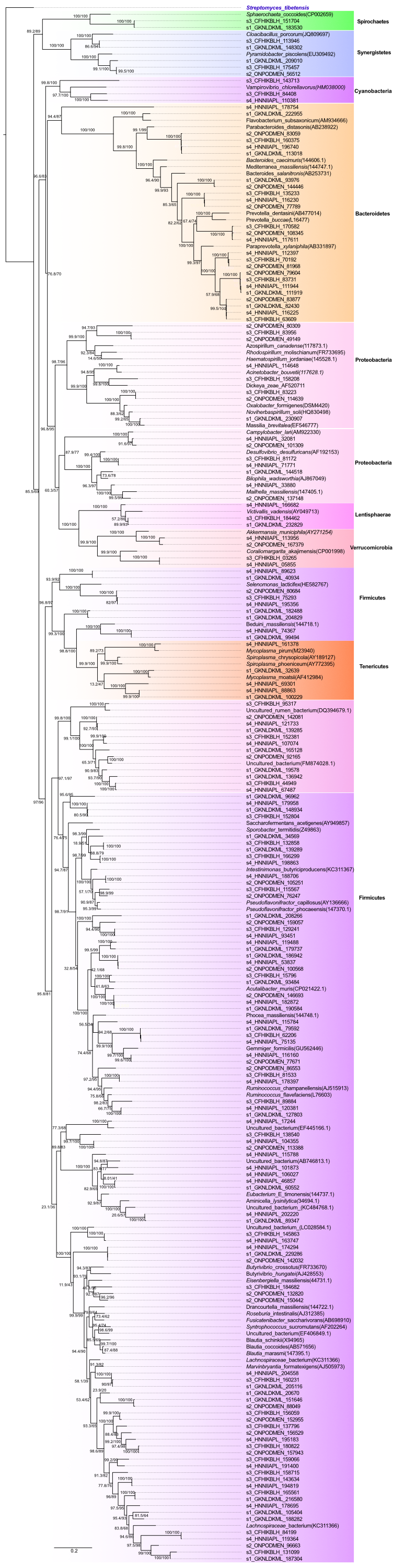

Supplement: Supplemental Information 2 — The labels s1_GKNLDKML, s2_ONPODMEN s3_CFHIKBLH and s4_HNNIIAPL correspond to each of the samples S1, S2, S3 and S4 from which they were obtained. We used Streptomyces 16S rRNA sequence as an outgroup [file peerj-09-11942-s002.png]
