# Supplementary material for: Diverse methanogens, bacteria and tannase genes in the feces of the endangered volcano rabbit (Romerolagus diazi)
Source: PeerJ. 2021 Aug 17;9:e11942. doi: 10.7717/peerj.11942 (PMC8378336; doi:10.7717/peerj.11942)
Supplement: Supplemental Information 3 [file peerj-09-11942-s003.docx]

| **Sample Name** | **Raw reads** | **Trimming** | **Number of contigs** |
| --- | --- | --- | --- |
| **S1** | 82.1 | 78.8 | 505,734 |
| **S2** | 76.2 | 71.6 | 423,407 |
| **S3** | 73.2 | 69.6 | 440,977 |
| **S4** | 101.2 | 96.6 | 383,582 |
