# Supplementary material for: Diverse methanogens, bacteria and tannase genes in the feces of the endangered volcano rabbit (Romerolagus diazi)
Source: PeerJ. 2021 Aug 17;9:e11942. doi: 10.7717/peerj.11942 (PMC8378336; doi:10.7717/peerj.11942)
Supplement: Supplemental Information 4 [file peerj-09-11942-s004.docx]

| **Taxa** | **Sample** | **N** | **SC** | **q0** | **q1** | **q2** |
| --- | --- | --- | --- | --- | --- | --- |
| Phyla Bacteria | S1 | 88552 | 100 | 29±0.01 | 4.7±0.04 | 3.2±0.02 |
|  | S2 | 54498 | 100 | 26±0.01 | 6.1±0.06 | 4.7±0.04 |
|  | S3 | 56761 | 100 | 28±0.01 | 6.1±0.06 | 4.3±0.04 |
|  | S4 | 55357 | 100 | 28±0.01 | 5.6±0.06 | 3.9±0.03 |
| Phyla Archaea | S1 | 869 | 100 | 3±0.01 | 2.3±0.1 | 2±0.1 |
|  | S2 | 446 | 100 | 3±0.01 | 1.7±0.1 | 1.4±0.1 |
|  | S3 | 25 | 100 | 2±0.01 | 2±0.1 | 2±0.2 |
|  | S4 | 29 | 100 | 2±0.01 | 2±0.1 | 2±0.2 |
| Genera Bacteria | S1 | 86230 | 100 | 669±0.1 | 130.9±1.6 | 39.3±0.7 |
|  | S2 | 51726 | 100 | 529±0.01 | 150.8±2.1 | 50.8±1.3 |
|  | S3 | 53793 | 100 | 594±0.01 | 172.4±2.4 | 60.1±1.3 |
|  | S4 | 52096 | 100 | 553±0.06 | 155.2±2.1 | 61±1.1 |
| Genera Archaea | S1 | 126 | 100 | 12±0.01 | 12±05 | 12±0.7 |
|  | S2 | 71 | 100 | 7±0.01 | 7±0.3 | 7±0.5 |
|  | S3 | 90 | 100 | 9±0.06 | 9±0.4 | 9±0.7 |
|  | S4 | 100 | 100 | 10±0.01 | 10±0.4 | 10±0.7 |
